# Disinfecting Power of Peroxide of Hydrogen

**Published:** 1890-10

**Authors:** 


### Disinfecting Power of Peroxide of Hydrogen ($H_2O_2$).

Dr. Altehofer, of Rostock, gives us in the *Cent. f. Bakteriologie und Parasitenkunde*, a careful study of the disinfecting power of this substance, for which he claims the especial merit that it does not alter the appearance, taste, or smell of water to which it has been added.

The first experiments were made with spring water, the second with hydrant water, and a third with river water. The first contained 560, the second 180, and the third, 1,800 germs in each cubic centimetre. To these several waters were added $H_2O_2$ in the proportion of 1:5000 and 1:10000, and after an exposure of 24 hours inoculations of gelatin and agar-agar were made. In each case abundant colonies developed, but those from the weaker solution were in greater number and more vigorous.

A second series was made upon hydrant water to which sewage had been added in the proportion of two per cent. and one-half per cent. The solutions were not sterilized even when $H_2O_2$ was added in the proportion of 1:2500.

Observations were made upon solutions containing $H_2O_2$ in the proportion of 1:1000. Three samples of potable water were used containing respectively, as was ascertained by cultivation, 160, 600, and 6,000 germs per cubic centimetre. The results in these three cases were, at the end of the seventh day, for the first sample three colonies, the second two, and the third ten. While this shows that the peroxide, in the proportion of 1:1000, is not sufficiently powerful to destroy all the ordinary germs of drinking water, it will destroy the greater number. Solutions of this strength impart a slight metallic taste to water, but this disappears shortly after it has been added. To 100 cubic centimetres of sterilized water one-half ccm. of sewage was added and one ccm. of $H_2O_2$. This mixture was found to be absolutely sterile at the end of twenty-four hours.

Experiments made with the typhoid and cholera bacillus showed that they were completely destroyed when $H_2O_2$ was present in the proportion of 1:1000.

The author recommends the disinfection of drinking water by this means in times of epidemics, not only from its efficiency and cheapness, but also because the potability of the water is not altered.

SINCE 1866, 202 women have received diplomas in France, of whom 35 were doctors of medicine, 67 bachelors of letters, 69 bachelors of science, 23 licentiates, and 2 pharmacists.

